# A novel *Rickettsia*, *Candidatus* Rickettsia takensis, and the first record of *Candidatus* Rickettsia laoensis in *Dermacentor* from Northwestern Thailand

**DOI:** 10.1038/s41598-023-37206-w

**Published:** 2023-06-21

**Authors:** Aummarin Chaloemthanetphong, Arunee Ahantarig, Dmitry A. Apanaskevich, Supanee Hirunkanokpun, Visut Baimai, Wachareeporn Trinachartvanit

**Affiliations:** 1 Department of Biology, Faculty of Science, Biodiversity Research Cluster, Mahidol University, Bangkok, 10400 Thailand; 2 Center of Excellence for Vectors and Vector-Borne Diseases, Faculty of Science, Mahidol University at Salaya, Nakhon Pathom, 73170 Thailand; 3 United States National Tick Collection, The James H. Oliver, Jr. Institute for Coastal Plain Science, Georgia Southern University, Statesboro, GA 30460-8056 USA; 4 Department of Biology, Faculty of Science, Ramkhamhaeng University, Bangkok, 10240 Thailand

**Keywords:** Phylogenetics, Bacteria, Microbiology, Molecular biology

## Abstract

Three hundred and forty-four tick samples were collected from vegetation at Taksin Maharat National Park, Tak province, northwestern Thailand. They were morphologically identified and molecularly confirmed by 16S rRNA and *COI* genes as *Dermacentor laothaiensis* (n = 105), *D. steini* (n = 139), and *D. auratus* (n = 100). These ticks were examined for the spotted fever group rickettsiae (SFGRs) using PCR and DNA sequencing of six genes; 17-kDa, *glt*A, 16S rRNA, *omp*A, *omp*B, and *sca*4. Of these ticks, 6.10% (21/344) gave positive results for the presence of SFGRs. Phylogenetic analyses of the SFGRs clearly indicated that a novel genotype assigned as *Candidatus* Rickettsia takensis was detected in *D. laothaiensis* (19/105) and at lesser frequency in *D. steini* (1/139). Furthermore, *Candidatus* Rickettsia laoensis was also found at a low frequency in *D. auratus* (1/100), the first record in Thailand. Although, the pathogenicities of these SFGRs remain unknown, our findings suggest potential risks of SFGRs being transmitted via ticks near the border between Thailand and Myanmar, a gateway of daily migrations of local people and visitors both legal and illegal.

## Introduction

Spotted fever group rickettsiae (SFGRs) belonging to the Order Rickettsiales are obligate intracellular Gram-negative bacteria; i.e. the bacteria need to associate with eukaryotic cells^1^. The link between SFGRs and emerging and re-emerging zoonosis infectious vector-borne diseases, is well recognized and studied worldwide^2,3^. At least thirty species of SFGRs have been reported over the past 35 years. Of these, 21 species have been identified as pathogens and others are of unknown pathogenicity^2,4,5^. Many species of rickettsiae were identified in ticks long before they were recognized as pathogenic agents in humans because the amount of *Rickettsia* DNA in arthropods is higher than in human blood^3^. Therefore, studies on species diversity of potential tick vectors of SFGRs are crucial to determine the presence or absence of any *Rickettsia* pathogens causing rickettsioses in humans.

At least five species of SFGRs (*R. conorii, R. helvetica, R. honei, R. japonica*, and *R. rhipicephali*) have been reported infecting humans in Southeast Asia^1,5^ although the incidence of spotted fever group rickettsioses varies across the region. The first four species listed above have been found in patients in Thailand^6–11^. Pathogenic *Rickettsia* species and other known rickettsial bacteria are associated with arthropod vectors, especially with hard ticks: these include *Ixodes granulatus*, *Dermacentor atrosignatus*, *Dermacentor* spp., *Haemaphysalis hystricis*, *Haemaphysalis* spp., and *Amblyomma* spp.^12–18^. In 2016, three divergent genotypes of SFGRs, namely *Ca.* R. laoensis and *Ca.* R. khammouanensis infecting *Haemaphysalis* sp., and *Ca.* R. mahosotii infecting *A. testudinarium* and *Haemaphysalis* sp. were reported in Laos but their pathogenicities were unknown^16^.

Identification of novel species of tick-borne rickettsiae has been greatly facilitated by DNA technology, especially gene sequence-based methods. Thus, molecular techniques are beneficial for the classification of *Rickettsia* as demonstrated by the description of the cultured *R. peacockii* based on genes 16S rDNA, *glt*A, and *omp*A sequence similarity, which was detected in *D. andersoni* from western Montana, USA^19^. Furthermore, a detailed study of *Rickettsia* using nucleotide sequence-based analysis of 5 genes (16S rRNA, *glt*A, *omp*A, *omp*B, and *sca*4) for classification of genus or species of SFGRs was proved to be a discriminative method for identification of the cultured *R. heilongjiangensis* found in *D. silvarum* from Heilongjiang, China^20^. In addition, Diop et al*.* used multilocus sequence typing (MLST) scheme methods to identify a novel *Rickettsia* species, *R. fournieri*, detected in *Argas lagenoplastis* ticks from Queensland, Australia^21^. Recently, Li et al*.* employed MLST to describe a new SFGR, *Ca.* R. xinyangensis, detected both in a patient and in *Haemaphysalis longicornis* ticks from Xinyang, China^22^.

In the present study, MLST was applied to evaluate the genetic polymorphisms and phylogenetic relationships of SFGRs detected in *Dermacentor* ticks residing in vegetation at Taksin Maharat National Park, Tak province, northwestern Thailand. The results indicated a novel genotype of *Rickettsia,* putatively named *Candidatus* Rickettsia takensis, detected in *D. laothaiensis* and *D. steini* ticks. The present report also provides the first record of *Ca.* R. laoensis infecting *D. auratus* in Thailand. We also present molecular evidence for species identifications of *D. laothaiensis, D. steini,* and *D. auratus.*

## Results

### Tick species identification

**Figure 1:**
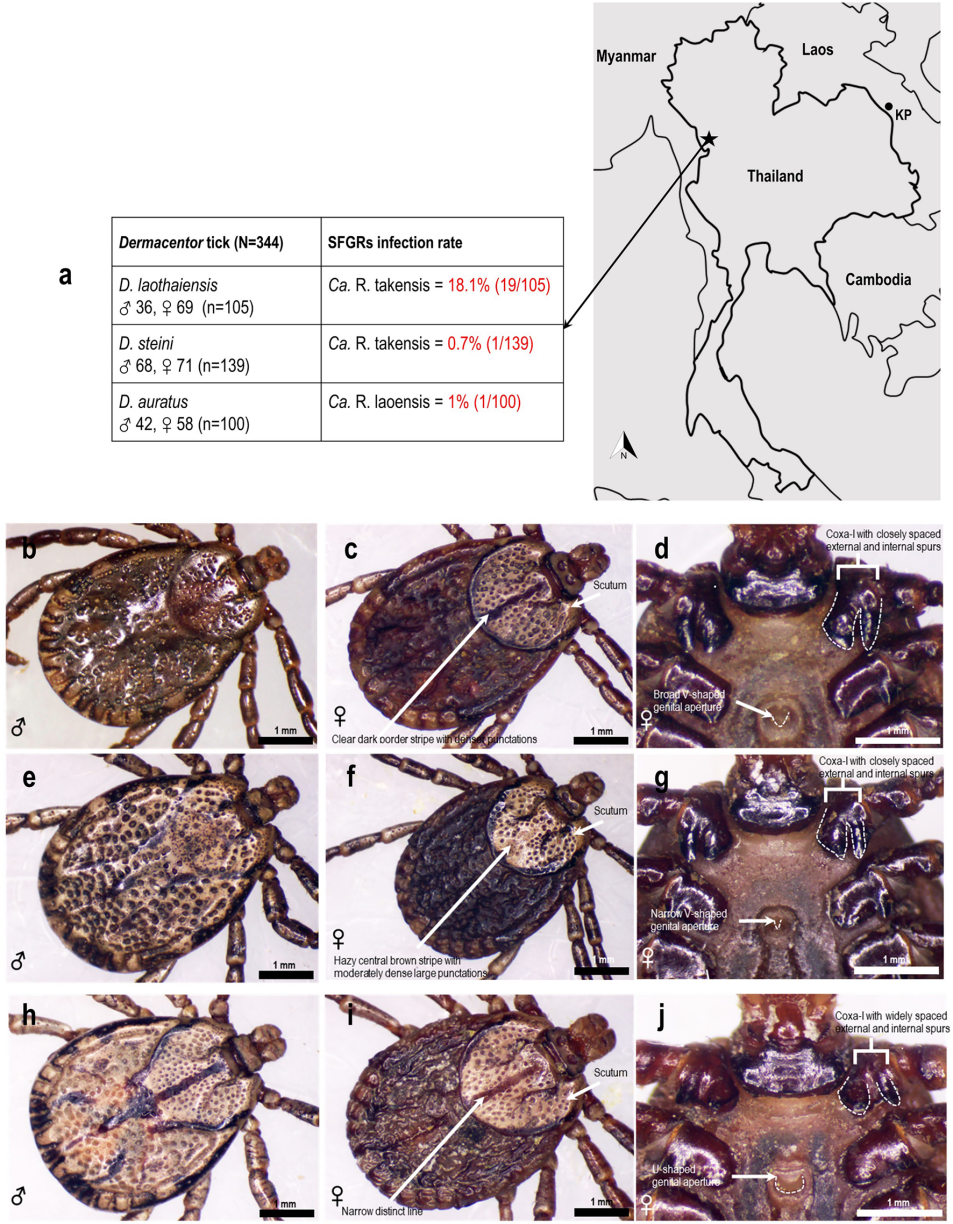
The location of the sampling site (black star) in Mae Sot district, Tak province, northwestern Thailand (neighboring Myanmar) (**a**). Indicated with a black dot, KP represents Khammouan province of Laos provided to enable a geographical comparison with Tak province of Thailand, where *Ca.* R. laoensis were detected. The numbers and species of *Dermacentor* tick with their SFGRs infection rates are provided. The photographic images of *D. steini* **(b–d)**, *D. laothaiensis* **(e–g)**, and *D. auratus* **(h–j)** tick morphologies are demonstrated with their different marked features. Figure 1 was designed and illustrated using PowerPoint 2019. (Photos: Aummarin Chaloemthanetphong; Map source: https://gistdaportal.gistda.or.th/portal/home/webmap/viewer.html?useExisting=1).

**Figure 2:**
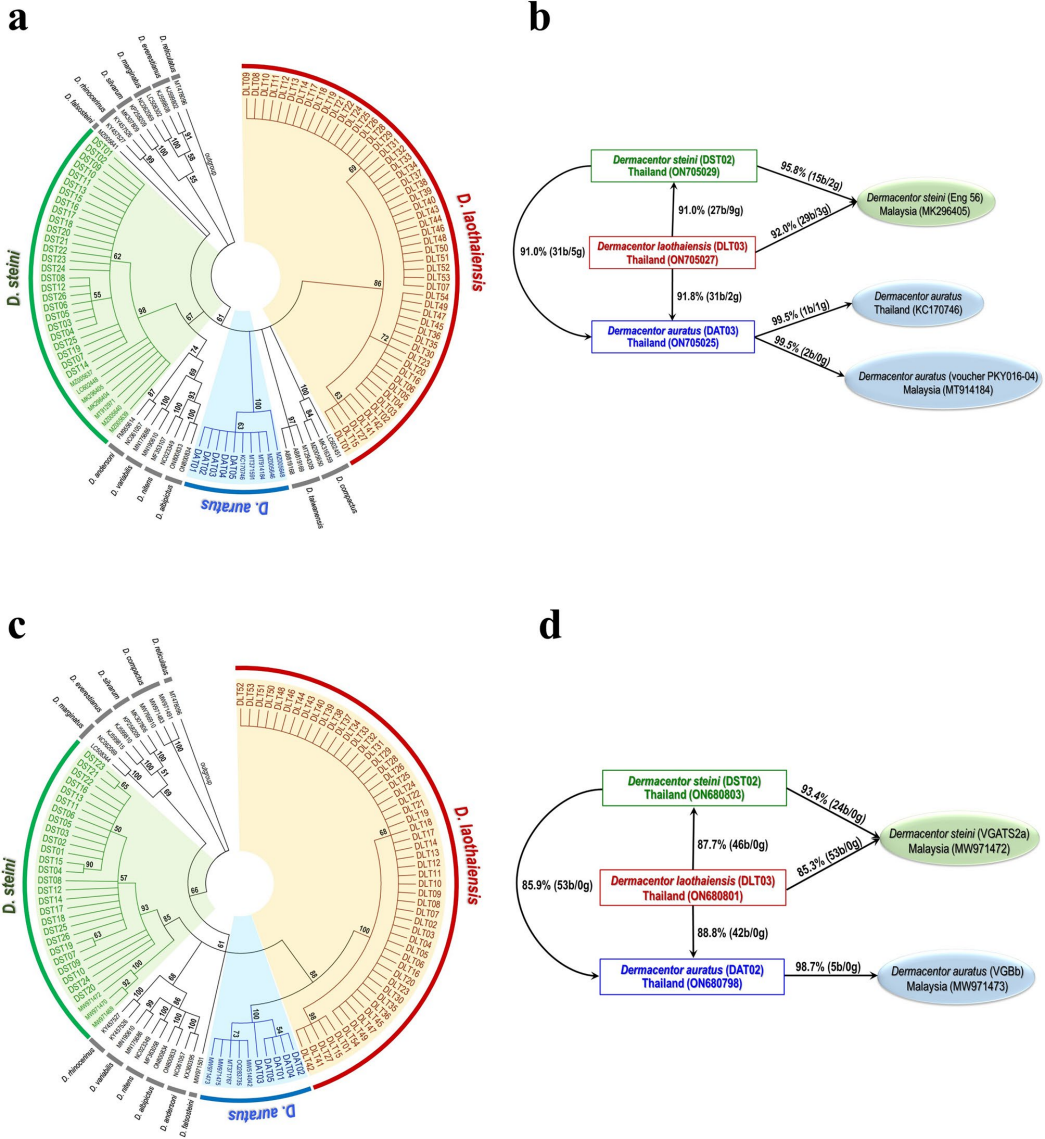
Phylogenetic analysis and the summary diagram of pairwise comparison among the tick species. Neighbor-joining trees based on partial gene sequences of the 16S rRNA (**a**) and *COI* (**c**) were constructed using the molecular evolutionary genetics analysis (MEGA) 7 software to represent three different species of the *Dermacentor* ticks from Tak province; *D. laothaiensis* (DLT), *D. steini* (DST), and *D. auratus* (DAT)*.* The reference species for *D. steini* and *D. auratus* are included with their accession numbers, while *D. laothaiensis* shows the first molecular identification*.* The outgroup was *D. reticulatus* (MT478096). Only bootstrap values > 50% are shown above each branch. The pairwise diagrams correspond to the 16S rRNA (**b**) and *COI* (**d**) with their similarity percentages and the number of base/gap differences (b/g). The diagrams of pairwise differences were designed and illustrated using PowerPoint 2019.

A total of 344 tick samples from the study area comprised three morphological species of *Dermacentor* ticks. These were *D. laothaiensis* (n = 105: 36 males, 69 females), *D. steini* (n = 139: 68 males, 71 females), and *D. auratus* (n = 100: 42 males, 58 females) (Fig. 1a). The morphology of *D. laothaiensis* was observed to be very similar to *D. steini*, and both were clearly different from *D. auratus* (Fig. 1b–j). *D. steini* showed closely spaced external and internal spurs on coxa-I, a distinct narrow dark border stripe on the densely punctuated scutum or pseudoscutum, and a narrowly to broadly V-shaped aperture of the female genital structure. *D. laothaiensis*, otherwise similar in morphology to *D. steini*, differed in having an indistinct broad hazy central brown stripe on the scutum or pseudoscutum, moderately dense large punctations, and very narrowly V-shaped genital aperture. *D. auratus* exhibited widely spaced external and internal spurs on coxa-I, a distinct narrow line through the center of scutum/ pseudoscutum, and a U-shaped genital aperture; Fig. 1b–j. Phylogenetic analysis results based on 16S rRNA and *COI* genes confirmed the existence of these 3 related species (Figs. 2a, c and S1). The pairwise diagram for these tick species based on 16S rRNA gene (Fig. 2b) demonstrated that *D. laothaiensis* had 91%, 91.8%, and 92% similarity compared with *D. steini* from Thailand (ON705029), *D. auratus* from Thailand (ON705025), and *D. steini* from Malaysia (MK296405), respectively*.* Their corresponding total numbers of differentiation positions of nucleotide base sequences alignments were 36 (27 bases/ 9 gaps), 33 (31 bases/ 2 gaps), and 32 (29 bases/ 3 gaps), respectively. Thailand *D. steini* was 91% similar to *D. auratus* from Thailand (ON705025) and 95.8% similar to *D. steini* from Malaysia (MK296405). It differed from both taxa in positions of nucleotide base sequences alignments by 36 (31 bases/ 5 gaps), and 17 (15 bases/ 2 gaps), respectively. Our *D. auratus* tick sample, showed a 99.5% match with both a *D. auratus* specimen originating from Malaysia (MT914184) and one other from Thailand (KC170746). Their total numbers of differentiation positions of nucleotide base sequences alignments were 2 (2 bases/ 0 gap) and 2 (1 base/ 1 gap), respectively. Likewise, the pairwise diagram for the tick species using the *COI* gene (Fig. 2d) demonstrated that *D. laothaiensis* was 87.7%, 88.8%, and 85.3% similar to *D. steini* from Thailand (ON680803), *D. auratus* from Thailand (ON680798) and a *D. steini* from Malaysia (MW971472). The total numbers of differentiation positions of nucleotide base sequences alignments were 46 (46 bases/ 0 gap), 42 (42 bases/ 0 gap), and 53 (53 bases/ 0 gap), respectively. While *D. steini* had 85.9% and 93.4% similarity compared with *D. auratus* from Thailand (ON680798) and *D. steini* from Malaysia (MW971472) and their total numbers of differentiation positions of nucleotide base sequences alignments were 53 (53 bases/ 0 gap) and 24 (24 bases/ 0 gap), respectively. Also, *D. auratus* (ON680798) had 98.7% similarity with *D. auratus* from Malaysia (MW971473), and the total numbers of differentiation positions of nucleotide base sequences alignments were 5 (5 bases/ 0 gap). All alignments of the related nucleotide bases and gap positions are shown in Fig. S2.

### Infection rate and molecular identification of SFGRs

**Figure 3:**
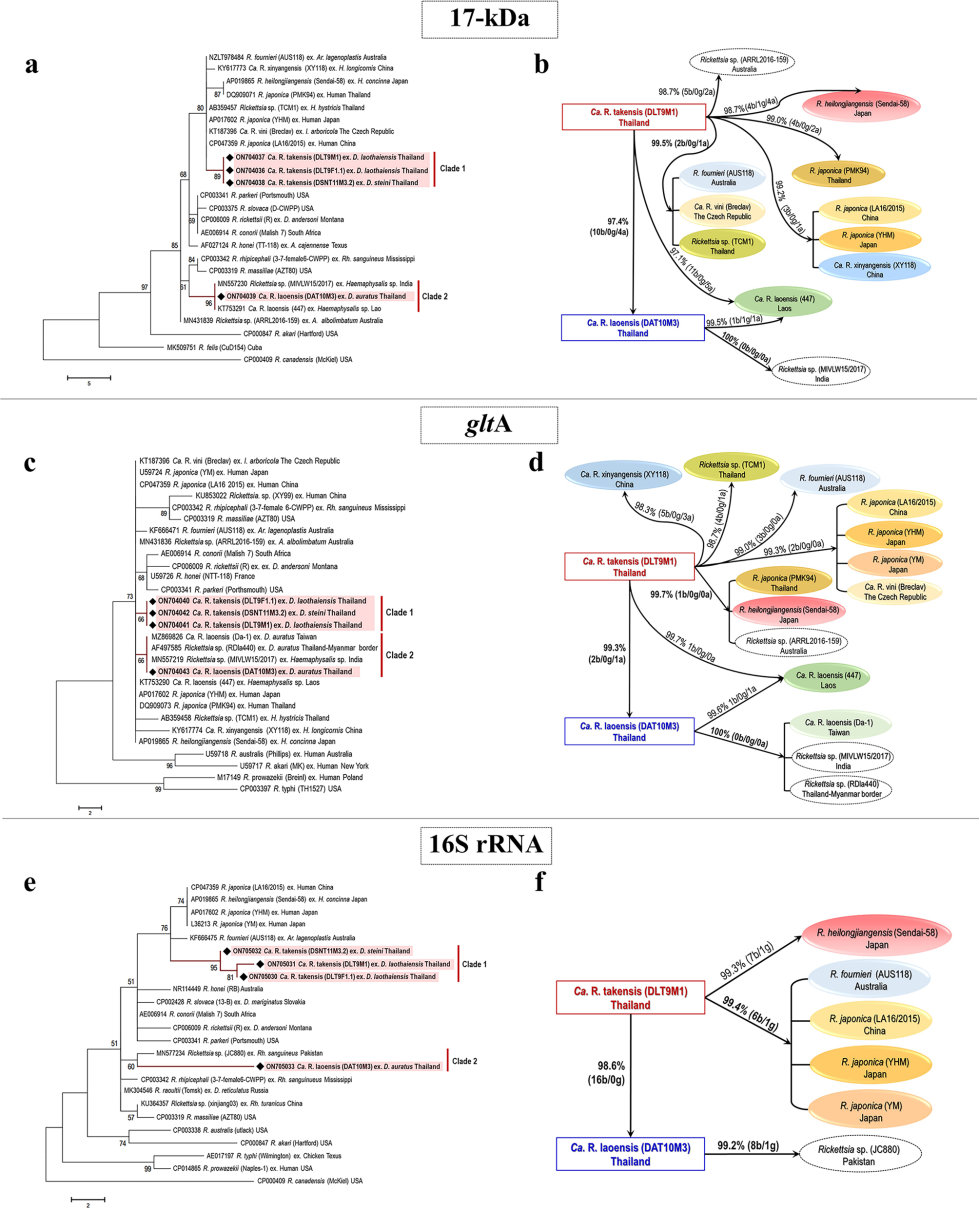

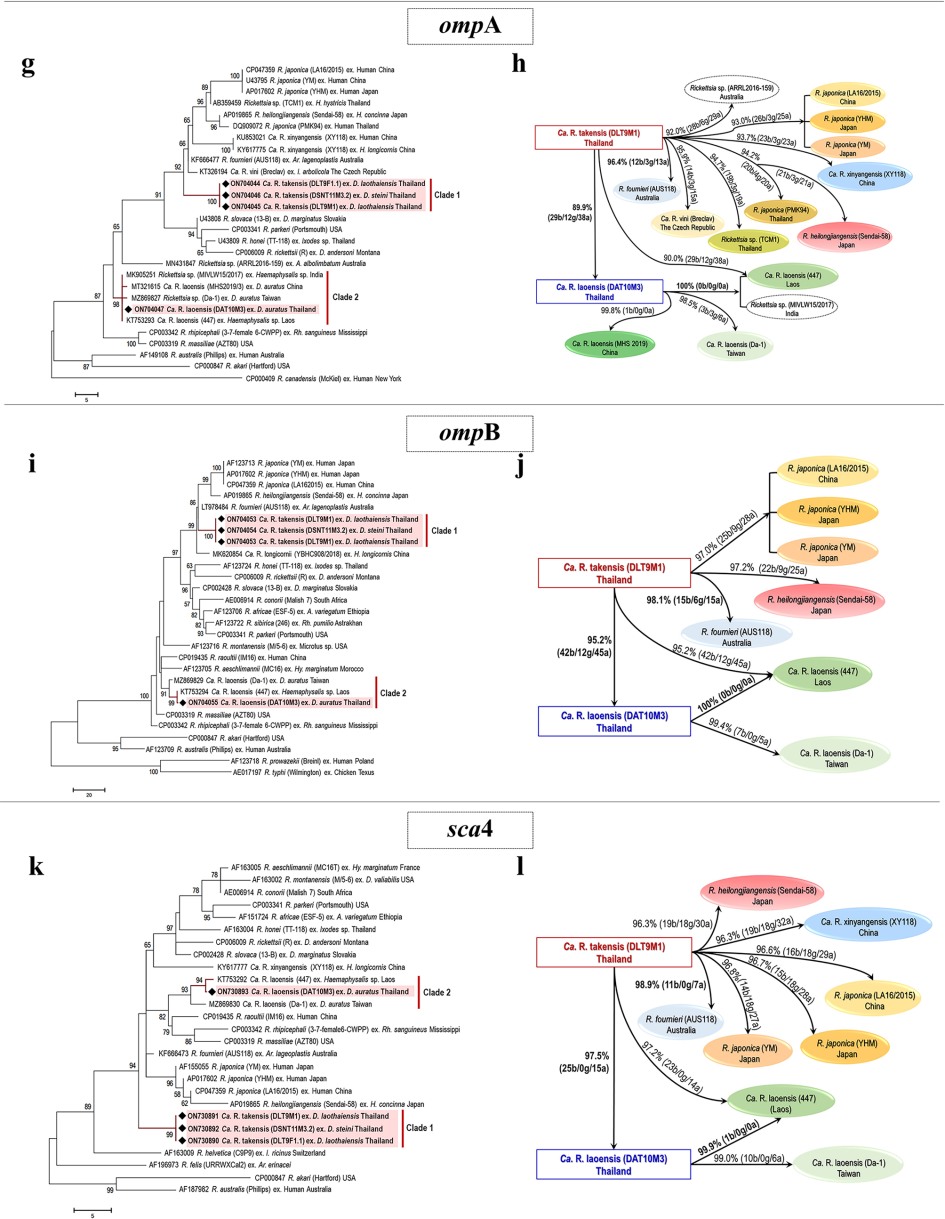
Phylogenetic analysis and the summary diagram of pairwise differences among *Rickettsia* species based on 17-kDa (**a,b**), *glt*A (**c,d**), 16S rRNA (**e,f**), *omp*A (**g,h**), *omp*B (**i,j**), and *sca*4 (**k,l**). The trees were constructed using the maximum-parsimony (MP) algorithms implemented in the molecular evolutionary genetics analysis (MEGA) 7 software. Bootstrap analysis was performed using the Subtree-Pruning-Regrafting (SPR) distances method (1,000 replications); only bootstrap values > 50% are shown. The black diamonds indicate the representative *Rickettsia* sequences of the samples in this study. The scale bar is in the units of substitution/site. The numbers in the summary diagram of pairwise differences among *Ca.* R. takensis*, Ca.* R. laoensis, and the references of related species of SFGRs indicate the percentages of nucleotide similarity and the number of base/gap/amino acid differences (b/g/a). The diagrams of pairwise differences were designed and illustrated using PowerPoint 2019.

The prevalence of rickettsiae from tick samples was investigated. The results using three housekeeping genes commonly used for SFGR screening, namely 17-kDa (434 bp), *glt*A (380 bp), and 16S rRNA (1,328 bp), all showed a positive rate of 6.10% (21/344). Further analyses of the amplified DNA samples employing three genes commonly used in *Rickettsia* genotyping, i.e., *omp*A (532 bp), *omp*B (1,295 bp), and *sca*4 (1,100 bp), clearly confirmed the presence of SFGRs in these positive ticks. The three species of ticks and their *Rickettsia* infection rates were as follows: 18.10% (19/105) in *D. laothaiensis*, 0.72% (1/139) in *D. steini*, and 1% (1/100) in *D. auratus* (Fig. 1). All positive amplicons were then sequenced and phylogenetic trees were constructed for the six individual targeted genes (17-kDa, *glt*A, 16S rRNA, *omp*A, *omp*B, and *sca*4). They showed more or less similar patterns of phylogenetic relationships with the closely related SFGRs from the NCBI database (Fig. 3). Our findings showed that there were two detected SFGRs in two separate clades as detailed below.

Clade 1, the isolated clade, included only our novel genotype, *Ca.* R. takensis. Based on 17-kDa, the novel genotype showed phylogenetic relationships with *R. japonica* from China and Japan, *Ca.* R. vini from the Czech Republic, *Rickettsia* sp. TCM1 from Thailand and *R. fournieri* from Australia (Fig. 3a). However, its *glt*A was closely related to *Rickettsia* sp. ARRL2016-159 from Australia, *R. japonica* from China and Japan, and *Ca.* R. vini from the Czech Republic (Fig. 3c). The 16S rRNA gene, *omp*A, *omp*B, and *sca*4 all demonstrated their closest relationships with *R. fournieri* from Australia (Fig. 3e,g,i,k).

Clade 2 included another SFGR detected in the present study, *Ca.* R. laoensis (DAT10M3). This may be the first Thailand record of *Ca.* R. laoensis. Based on the 17-kDa gene, the Thailand specimen grouped with *Ca.* R. laoensis from Laos and *Rickettsia* sp. from India (Fig. 3a). Its *glt*A gene grouped with *Rickettsia* sp. from India, *Rickettsia* sp. RDla440 from the Thailand-Myanmar border and *Ca.* R. laoensis from Taiwan (Fig. 3c). On 16S rRNA*,* it grouped with *Rickettsia* sp. from Pakistan (Fig. 3e). Close phylogenetic relationships with *Ca.* R. laoensis were notably demonstrated for *omp*A from India and Laos (Fig. 3g), *omp*B and *sca*4 from Laos (Fig. 3i,k).

The results of these phylogenetic analyses supports the conclusion that there may be two unknown groups of *Rickettsia* spp. occurring in these tick vectors, which are tentatively referred to *Ca.* R. takensis and *Ca.* R. laoensis as mentioned above. However, some other SFGRs also showed phylogenetic relationships close to our discovered SFGRs. Therefore, to validate the species classification of our two putative detected SFGRs, their sequence alignments, and pairwise comparisons were performed against reference SFGRs. The results of the nucleotide similarity percentages determined were then applied to pose criteria for a novel *Rickettsia* determination using gene sequences^20^. The details of the nucleotide bases and amino acid variations are demonstrated in Figs. 3 and S3.

The highest percentage, indicating the closest similarity in each target gene is shown in bold font in the diagram. The 17-kDa gene which was used to screen *Rickettsia* in the population, showed the highest value (99.5%; 2 bases/ 0 gap/ 1 amino acid) for several SFGRs (*R. fournieri* from Australia, *Ca.* R. vini from the Czech Republic, and *Rickettsia* sp. TCM1 from Thailand; Fig. 3b). The *glt*A gene showed the highest value of similarity (99.7%, 1 base/ 0 gap/ 0 amino acid) for *R. japonica* from Thailand (PMK94), *R. heilongjiangensis* from Japan, and *Rickettsia* sp. ARRL2016-159 from Australia (Fig. 3d). For 16S rRNA the highest values of 99.4% (6 bases/ 1 gap) were obtained for *R. fournieri* from Australia, *R. japonica* from China (LA16/2015) and Japan (YHM and YM) (Fig. 3f). The *omp*A, *omp*B, and *sca*4 genes demonstrated similar results, finding that our *Ca.* R. takensis was closest to the *R. fournieri* from Australia. Their highest percentages and detail of variables were as follows: *omp*A 96.4% (12 bases/ 3 gaps/ 13 amino acids) (Fig. 3h), *omp*B 98.1% (15 bases/ 6 gaps/ 15 amino acids) (Fig. 3j), and *sca*4 98.9% (11 bases/ 0 gap/ 7 amino acids) (Fig. 3l). In addition, the pairwise comparisons between *Ca.* R. takensis and either *Ca.* R. laoensis (DAT10M3) from Thailand or *Rickettsia* sp. RDla440 from the Thailand-Myanmar border, showed exact similarity of 99.3% (2 bases/ 0 gap/ 1 amino acid) based on the only data available for the *glt*A gene (Fig. 3d). Based on the criteria for a novel *Rickettsia* determination using gene sequences of 16S rRNA, *glt*A, *omp*A, *omp*B, and *sca*4, an isolate can be classified as a new *Rickettsia* genotype when it exhibits no more than one of the following degrees of nucleotide similarity with the most homologous validated species (cutoff percentages ≥ 99.8, ≥ 99.9, ≥ 98.8, ≥ 99.2, and ≥ 99.3)^20^. The result of *Ca.* R. takensis fulfilled this criterion with the degrees of similarity 99.4, 99.7, 96.4, 98.1, and 98.9, respectively. None of the degrees were equal to or over the cutoff percentages of the standard criteria. This indicated the occurrence of a new *Rickettsia* genotype. Moreover, this *Rickettsia* was detected in ticks, not from rickettsial isolates: therefore *Candidatus* status could be applied. Therefore, this evidence showed that we found a novel genotype of SFGR in *D. laothaiensis* and *D. steini* tick samples. In addition, this novel *Rickettsia* was assigned as *Candidatus* Rickettsia takensis after the name of the collecting site, Tak province.

According to the evidence, we also found *Ca.* R. laoensis (DAT10M3) infection in one male in the *D. auratus* tick samples. The highest percentages of similarities based on the targeted genes were shown in pairwise comparisons. (1) DAT10M3 and *Ca.* R. laoensis from Laos *omp*A 100%, *omp*B 100%, and *sca*4 99.9% (1 base/ 0 gap/ 0 amino acid) (Fig. 3h, 3j, and 3l). (2) DAT10M3 and *Ca.* R. laoensis from China *omp*A 99.8% (1 base/ 0 gap / 0 amino acid) (Fig. 3h). (3) DAT10M3 and *Rickettsia* sp. from India 17-kDa 100%, *glt*A 100%, and *omp*A 100% (Fig. 3b,d,h). (4) DAT10M3 compared with either *Rickettsia* sp. from the Thailand-Myanmar border or *Ca.* R. laoensis from Taiwan *glt*A 100% (Fig. 3d). (5) DAT10M3 and *Rickettsia* sp. JC880 from Pakistan 16S rRNA 99.2% (8 bases/ 1 gap) (Fig. 3f). Overall, phylogenetic analyses and sequence alignments indicated that DAT10M3 and *Ca.* R. laoensis from Laos shared almost identical genotypes. This shows that *Ca.* R. laoensis is present on the northwestern border of Thailand. However, there have not yet been any reports on whether *Ca.* R. laoensis is a pathogen although it was detected in ticks (*Haemaphysalis* sp.^16^ and *D. auratus* in the present study).

## Discussion

More than 40 species of *Dermacentor* ticks are known worldwide^23–25^. So far, eight species (*D. auratus, D. compactus, D. steini, D. filippovae, D. pasteuri, D. laothaiensis*, *D. falsosteini*, and *D. tricuspis*) have been found in Thailand^24–28^. Of these, three species were morphologically and molecularly identified as *D. laothaiensis, D. steini,* and *D. auratus* in the present study. *D. laothaiensis* was recently described, and it was reported as morphologically closely related to *D. steini*^26^. However, we found that *D. laothaiensis* was quite distinct from *D. steini* as these two species resolving into separate clades during phylogenetic analyses. The distinction was further supported as data was harvested from nucleotide sequence alignments (Fig. S2) demonstrating differentiation during pairwise comparisons among the tick species (Fig. 2). The results of our molecular analyses and phylogenetic relationships based on 16S rRNA and *COI* genes constitute the first molecular evidence indicating the difference between the *D. laothaiensis* and *D. steini*, and also showed that both were distinct from *D. auratus*.

In Thailand, several studies have examined the detection of *Rickettsia* in *Dermacentor* ticks collected from vegetation and mammal hosts. Malaisri et al*.* obtained 13 adult-stage *Dermacentor* ticks from vegetation and observed no evidence of rickettsial infection^29^. Sumrandee et al*.* collected a total of 16 *Dermacentor* ticks (15 adults and 1 nymph) from Artiodactyla and identified rickettsial infection in 5 of them, accounting for 31.3% (5/16)^30^. Nooroong et al. (2018) collected 114 adult *Dermacentor* ticks from vegetation, of which 5 were infected with *Rickettsia*, representing 4.4% (5/114)^31^. Takhampunya et al*.* investigated 58 *Dermacentor* ticks, consisting of 35 larvae and 23 adults, and detected rickettsial infection in only 1 adult, accounting for 1.7% (1/58)^32^. Finally, Hirunkanokpun et al*.* examined 8 *Dermacentor auratus* nymphs from a Burmese ferret badger but found no rickettsial infection in these nymphs^18^. These studies indicate a rare or relatively low incidence of rickettsial infection in *Dermacentor* ticks, except for the findings of Sumrandee et al*.* with a rate of 31.3%^30^.

Reviews of SFGRs in Thailand^1^ have previously revealed at least 5 defined species (*R. honei*, *R. japonica*, *R. helvetica*, *R. rickettsii*, and *R. thailandii*). In addition, another 4 SFGRs were also reported as genotypically related to *R. raoultii*, *R. tamurae*, *R. monacensis*, and *R. montana*^1,29–34^. Globally distributed SFGRs comprise more than 20 species^3^of which over 40% are likely found in Thailand. However, only a few studies have been conducted there compared to other regions. Interestingly, several SFGRs were reported from the Thailand-Myanmar border. In 1994, SFGR antibodies for *R. japonica* and *R. honei* (TT-118) were detected in rat serum collected in the 1970s at Kanchanaburi^6^ (14°N 99°E). In 2003, *Rickettsia* sp. RDa420 infecting *D. auratus* and *Rickettsia* sp. RDla440 infecting *Dermacentor* larvae were also reported from Kanchanaburi^12^. In the present study, there were two different genotypes of SFGRs, one of which was proposed as a novel genotype, *Ca.* R. takensis, and the other provided Thailand’s first record of *Ca.* R. laoensis in *Dermacentor* ticks. Both were collected from our study area in Tak (16°N 99°E), another border province with the same longitude comparing with Kanchanaburi province as mentioned above that there were some reports of SFGRs. This may indicate the distribution of SFGRs along the Thailand-Myanmar border.

In this study, our phylogenetic classification of *Ca*. R. takensis lacked clarity because its genotype showed close relationships to (1) *R. fournieri* from Australia and *R. japonica* from China and Japan (16S rRNA) (2) *Rickettsia* sp. from Australia, *R. japonica* from Thailand, and *R. heilongjiangensis* from Japan (*glt*A) and (3) *R. fournieri* from Australia (*omp*A, *omp*B, and *sca*4) (Fig. 3d,f,h,j,l). However, individually targeted gene trees demonstrated a unique *Rickettsia* genotype resolving separately from the other reference clades (Fig. 3a,c,e,g,i,k). This was likely supported by the results summarized in the diagrams of Fig. 3. The criteria for classifying *Rickettsia* species^20^ were applied, and it was confirmed that *Ca.* R. takensis was a member of the genus *Rickettsia* according to its 16S rRNA and *glt*A homology against some other validated *Rickettsia* spp. by the degree of homology of ≥ 98.1% and ≥ 86.5%, respectively for these two genes. Furthermore, the presence of the *omp*A gene also confirmed that it belonged to the SFGRs. Again, when we examined *Rickettsia* species identification by MLST, we found that in no case was the degree of nucleotide similarity (99.4, 99.7, 96.4, 98.1, and 98.9), compared to the most homologous species from the NCBI database (Fig. 3 and Table 1), equal to or over the respective standard criteria cutoff percentages (≥ 99.8, ≥ 99.9, ≥ 98.8, ≥ 99.2, and ≥ 99.3). Therefore, these results indicated the uniqueness of a novel genotype of *Ca.* R. takensis reported in this study. Moreover, current investigations of rickettsiae associated with *Dermacentor* ticks in the vicinity of the Thailand-Myanmar border showed the presence of a novel genotype, *Ca.* R. takensis, predominantly detected in *D. laothaiensis* and to a lesser extent in *D. steini*. *Ca*. R. takensis found in all 20 ticks exhibited similar nucleotide sequences for the six genes (17-kDa, *glt*A, 16S rRNA, *omp*A, *omp*B, and *sca*4) examined. Surprisingly, only one male of *D. auratus* was infected with *Ca.* R. laoensis (DAT10M3). Pairwise comparisons of nucleotide sequences of these two genotypes of rickettsiae based on the targeted genes showed 97.4%, 99.3%, 98.6%, 89.9%, 95.2%, and 97.5% similarities, respectively. These data confirmed that our detected SFGRs, either *Ca.* R. takensis or *Ca.* R. laoensis, were two distinct *Rickettsia* species that infected the population of our tick samples.

**Table 1:** Summary data.

Ref.* Rickettsia* from GenBank	17-kDa (no % cutoff)		*glt*A (99.9%cutoff)		16S rRNA (99.8%cutoff)		*omp*A (98.8%cutoff)		*omp*B (99.2%cutoff)		*sca*4 (99.3%cutoff)	
	%nucleotide similarity (matched/total number of nucleotide)	Pairwise differences for # nucleotide base/gap /amino acid	%nucleotide similarity (matched/total number of nucleotide)	Pairwise differences for # nucleotide base/gap /amino acid	%nucleotide similarity (matched/total number of nucleotide)	Pairwise differences for # nucleotide base/gap	%nucleotide similarity (matched/total number of nucleotide)	Pairwise differences for # nucleotide base/gap /amino acid	%nucleotide similarity (matched/total number of nucleotide)	Pairwise differences for # nucleotide base/gap /amino acid	%nucleotide similarity (matched/total number of nucleotide)	Pairwise differences for # nucleotide base/gap /amino acid
	*Ca.* R. takensis											
*R. fournieri* (AUS118) Australia	**99.5 (383/385)**	2/0/1	99.0 (296/299)	3/0/0	**99.4 (1,128/1,135)**	6/1	**96.4 (397/412)**	12/3/13	**98.1 (1,099/1,120)**	15/6/15	**98.9 (982/993)**	11/0/7
*R. japonica* (LA16/2015) China	99.2 (382/385)	3/0/1	99.3 (297/299)	2/0/0	**99.4 (1,128/1,135)**	6/1	93.0 (383/412)	26/3/25	97.0 (1,086/1,120)	25/9/28	96.6 (959/993)	16/18/29
Ca. *R. vini* (Breclav) The Czech Republic	**99.5 (383/385)**	2/0/1	99.3 (297/299)	2/0/0	NA	NA	95.9 (395/412)	14/3/15	NA	NA	NA	NA
*Rickettsia* sp. (ARRL2016-159) Australia	98.7 (380/385)	5/0/2	**99.7 (298/299)**	1/0/0	NA	NA	92.0 (378/412)	28/6/29	NA	NA	NA	NA
Ca. *R. xinyangensis* (XY118) China	99.2 (382/385)	3/0/1	98.3 (294/299)	5/0/3	NA	NA	93.7 (386/412)	23/3/23	NA	NA	96.3 (956/993)	19/18/32
*R. japonica* (YHM) Japan	99.2 (382/385)	3/0/1	99.3 (297/299)	2/0/0	**99.4 (1,128/1,135)**	6/1	93.0 (383/412)	26/3/25	97.0 (1,086/1,120)	25/9/28	96.7 (960/993)	15/18/28
*R. japonica* (PMK94) Thailand	99.0 (381/385)	4/0/2	**99.7 (298/299)**	1/0/0	NA	NA	94.2 (388/412)	20/4/20	NA	NA	NA	NA
*R. japonica* (TCM1) Thailand	**99.5 (383/385)**	2/0/1	98.7 (295/299)	4/0/1	NA	NA	94.7 (390/412)	19/3/19	NA	NA	NA	NA
*R. japonica* (YM) Japan	NA	NA	99.3 (297/299)	2/0/0	**99.4 (1,128/1,135)**	6/1	93.0 (383/412)	26/3/25	97.0 (1,086/1,120)	25/9/28	96.8 (961/993)	14/18/27
*R. heilongjiangensis* (Sendai-58) Japan	98.7 (380/385)	4/1/4	**99.7 (298/299)**	1/0/0	99.3 (1,127/1,135)	7/1	94.2 (388/412)	21/3/21	97.2 (1,089/1,120)	22/9/25	96.3 (956/993)	19/18/30
	*Ca.* R. laoensis											
*Ca.* R. laoensis (447) Laos	99.5 (383/385)	1/1/1	99.6 (258/259)	1/0/1	NA	NA	**100 (412/412)**	0/0/0	**100 (1,120/1,120)**	0/0/0	**99.9 (809/810)**	1/0/0
*Ca.* R. laoensis (MHS 2019/3) China	NA	NA	NA	NA	NA	NA	99.8 (411/412)	1/0/0	NA	NA	NA	NA
*Ca.* R. laoensis (Da-1) Taiwan	NA	NA	**100 (299/299)**	0/0/0	NA	NA	98.5 (406/412)	3/3/6	99.4 (1,113/1,120)	7/0/5	99.0 (893/993)	10/0/6
*Rickettsia* sp. (MIVLW15/2017) India	**100 (383/385)**	0/0/0	**100 (299/299)**	0/0/0	NA	NA	**100(412/412)**	0/0/0	NA	NA	NA	NA
*Rickettsia* sp. (RDla440) Thailand-Myanmar border	NA	NA	**100 (299/299)**	0/0/0	NA	NA	NA	NA	NA	NA	NA	NA
*Rickettsia* sp. (JC880) Pakistan	NA	NA	NA	NA	**99.2 (1,126/1,135)**	8/1	NA	NA	NA	NA	NA	NA
*Ca.* R. takensis (DLT9M1) Thailand	97.4 (375/385)	10/0/4	99.3 (297/299)	2/0/1	98.6 (1,119/1,135)	16/0	90.0 (371/412)	29/12/38	95.2 (1,066/1,120)	42/12/45	97.5 (968/993)	25/0/15

The percentages of nucleotide sequence similarity and the variations of nucleotide bases, gaps, and amino acids are shown. The pairwise comparisons were done among amplicons of either the *Ca.* R. takensis or the *Ca.* R. laoensis with the reference sequences of the related rickettsiae from the GenBank for each target gene (17-kDa, *glt*A, 16S rRNA, *omp*A, *omp*B, and *sca*4). Bold text indicates the highest similarity percentages with the partial sequence size in parentheses. *NA* not available.

Another differentiated SFGRs genotype, *Ca.* R. laoensis (DAT10M3), was classified and this yielded the first molecular evidence for its presence in Thailand. Importantly, based on the *gltA* gene, our sample DAT10M3 exhibited a close relationship with the *Rickettsia* sp. RDla440, the SFGRs reported in *Dermacentor* larvae from Kanchanaburi^12^. However, the *gltA* gene sequence analysis was the only available data for RDla440, although it yielded 100% similarity compared to our DAT10M3 (299 bp) sequence alignment. Subsequently it was reported that RDla440 appeared to be more closely related to the sequences of *Rickettsia* sp. strain DnS14 and *Rickettsia* sp. strain RpA4, differing in only 2 bp (99.7% similarity) based on *gltA* (1,108 bp)^12^. However, both DnS14 and RpA4 were later identified as *R. raoultii* based on 16S rRNA, *glt*A, *omp*A, *omp*B, and *sca*4 genes^35^. Therefore, although the results with the *gltA* gene indicated DAT10M3 had 99.7% similarity (variable of 1 base) with either DnS14 or RpA4 strains detected in ticks from the former Soviet Union^36^, data from a single gene might be insufficient for SFGRs classification. Likewise, based on our results, our sample DAT10M3 was most likely *Ca.* R. laoensis, related to samples of *Ca.* R. laoensis reported in Laos^16^. However, it was not certain whether the RDla440 detected earlier at the same border longitude of this was in fact *Ca.* R. laoensis due to the limited molecular evidence. Although *glt*A has frequently been used as a target for generic diagnostics based on PCR because this approach can easily identify a number of *Rickettsia*^37–39^, this gene is also highly conserved. Therefore, *glt*A on its own might not be enough to differentiate between closely related *Rickettsia* species. Consequently, the MLST of 17-kDa, *glt*A, 16S rRNA, *omp*A, *omp*B, and *sca*4 were suggested as potentially helpful and appropriate for the reconstruction of the evolutionary relationship of diverse but closely related *Rickettsia* species, as documented in our results and previous reports^1,2,16,38,40,41^.

*Ca.* R. laoensis infecting *Haemaphysalis* nymphs (4.5%) was first reported in Khammouan province, Laos^16^. We found that *Ca.* R. laoensis in Thailand and Laos showed the highest percentage values during pairwise comparison of the five genes, 17-kDa, *glt*A, *omp*A, *omp*B, and *sca*4, with 99.5%, 99.6%, 100%, 100%, and 99.9% similarity, respectively. Khammouan province in Laos is located near the northeastern border of Thailand, bordering the Mekong River, about 700 km east of Tak province which lies on the Myanmar border (Fig. 1). This geographical evidence seems to suggest that *Ca.* R. laoensis is probably a common species, and widely distributed in Thailand, at least in the upper part of the country. In addition, Wang et al. (2020) found *Ca.* R. laoensis infecting 4 species of *Haemaphysalis* (*H. bispinosa*, *H. longicornis*, *H. flava*, and *H. hystricis*) and 4 species of *Dermacentor* (*D. auratus*, *D. atrosignatus*, *D. silvarum*, and *D. taiwanensis*) in eastern China, based on the nucleotide sequence of *omp*A gene (MT321615)^42^. These authors also showed that *Ca.* R. laoensis in China was molecularly similar to the *Rickettsia* sp. (MK905251) infecting *Haemaphysalis* ticks in the Western Ghats, India (Arunkumar, G., GenBank in NCBI, 2019). Although, the nucleotide sequences of 3 genes (17-kDa, *glt*A, and *omp*A) showed that *Ca.* R. laoensis from Thailand and India have close relationship (Table 1). And recently, Yen et al*.* (2022) also reported *Ca.* R. laoensis infecting *D. auratus* in Taiwan, based on nucleotide sequences of four genes; *glt*A (MZ869826), *omp*A (MZ869827), *omp*B (MZ869829), and *sca*4 (MZ869830)^41^. Taken together, these data suggest that *Ca.* R. laoensis from Thailand, Laos, China, Taiwan, and India may represent a complex of species within this *Rickettsia* cluster. This complex cluster reflects species diversity of SFGRs in Asia, centered around Southeast Asia, including Thailand. As well as seeking to detect *Ca.* R. laoensis in different species of tick vectors in this region, more information about its pathogenicity is needed. Therefore, more fieldwork investigating *Ca.* R. laoensis infection in ticks and humans in this region is required. Although the information on species diversity of rickettsiae related to the emerging rickettsioses via tick vectors is still poorly understood in Thailand, our study has enhanced knowledge of SFGRs harbored in these 3 species of *Dermacentor* ticks in this particular area of northwestern Thailand. Thus, the prevalence of these SFGRs infected tick vectors along the border of Thailand and Myanmar, extending for approximately 2,400 km from the north to the south, will be very useful and informative because these areas are gateways for the movement of local people between the two countries. The border areas also involve legal and illegal livestock trading, increasing the potential risk of SFGRs exposure in this region.

Furthermore, the polymorphic genotypes of *Ca.* R. takensis and *Ca.* R. laoensis discovered in this study indicated the genetic diversity of SFGRs in this area. In addition, understanding the movements of wildlife, especially mammalian hosts of ticks, may contribute to the knowledge of the distribution of rickettsial agents in this particular forested area. The data seem to suggest a potential risk for the distribution of this SFGRs in neighboring countries, including Thailand, Laos, and Myanmar. The diagnosis and management of rickettsial infections in this region remains challenging, for instance, the results of clinical differential diagnoses for SFGRs are usually disregarded in resource-poor settings because of the limitations of immunofluorescence serologic tests caused by antigenic cross-reactions^43^. While the identification of phylogenetically distinct SFGRs to species by molecular methods is well known to have practical applications, public health facilities and attention related to rickettsiae needs to be improved to enable greater accessibility in Southeast Asia.

## Materials and methods

### Tick collection and species identification

A total of 344 adults of *Dermacentor* ticks were collected from vegetation in the forest of a tourist trail (with shrubs and trees along both sides) at Taksin Maharat National Park, Mae Sot district, Tak province, about 20 km from the Thailand-Myanmar border (Fig. 1a). The collecting of tick samples was conducted upon approval from the Department of National Parks, Wildlife and Plant Conservation of Thailand (permission no. TS 0907.4/10,680). Collections were made at 754 m asl, and ticks were searched for and collected, with blunt forceps or hand picking from vegetation in 2016 with an average temperature of 23 °C, and an average humidity of 80% for the environmental conditions. All tick samples were kept in 70% ethanol and stored at −20 °C for laboratory study. Each tick was morphologically identified using available descriptions^26,44,45^. The samples of these ticks were subsequently confirmed by molecular techniques using DNA sequencing of two genes; 16S ribosomal RNA (16S rRNA: 16S + 1, 16S-1) and *cytochrome c oxidase I* (*COI*: RON, TCOIR), as described by Black and Piesman and Simon et al*.*^46,47^. The references for primers used are listed in Table 2.

**Table 2:** Oligonucleotide primers used in this study.

Target gene	Primer	Sequences (5’-3’)	Amplicon (bp)	References
Tick identification				
16S rRNA	16S + 1	CTGCTCAATGATTTTTTAAATTGCTGTGG	460	^46^
	16S-1	CCGGTCTGAACTCAGATCAAGT		
*COI*	RON	GGAGCYCCWGATATAGCTTTCCC	463	^47^
	TCOIR	WGGRTGRCCAAARAATCAAAATA		
SFGRs identification				
17-kDa	Rr17.61p	GCTCTTGCAACTTCTATGTT	434	^48^
	Rr17.492n	CATTGTTCGTCAGGTTGGCG		
16S rRNA	Rick-16SF3	ATCAGTACGGAATAACTTTTA	1,328	^49^
	Rick-16SF4	TGCCTCTTGCGTTAGCTCAC		
*glt*A	RpCS.887p	GGGGGCCTGCTCACGGCGG	380	^50^
	RpCS.1258n	ATTGCAAAAAGTACAGTGAAC		
*omp*A	Rr190.70p	ATGGCGAATATTTCTCCAAAA	532	
	Rr190.602n	AGTGCAGCATTCGCTCCCCCT		
*omp*B	120-607F	AATATCGGTGACGGTCAAGG	1,295	^51^
	120–1902	CCGTCATTTCCAATAACTAACTC		
	OF	GTAACCGGAAGTAATCGTTTCGTAA	503	^22^
	OR	GCTTTATAACCAGCTAAACCACC		
*sca*4	RrD749F	TGGTAGCATTAAAAGCTGATGG	1,100	^20^
	RrD1826R	TCTAAATKCTGCTGMATCAAT		

### DNA extraction

DNA extractions of all tick samples were performed after they had been morphologically identified. An individual tick was serially rinsed and cleaned thoroughly in 70% ethanol, 10% sodium hypochlorite (NaOCl), and sterilized distilled water three times (1 min each) to avoid external bacterial contamination. The total DNA of each tick sample was extracted from the whole body of individual adults using the QIAamp DNA Extraction Mini Kit for Tissue (QIAGEN, Germany) according to the manufacturer's instructions. Then, 16S rRNA amplification was used to monitor the DNA extraction quality before the product was stored at − 20 °C for further study.

### Rickettsia detection, identification and sequencing analyses

PCR was performed to amplify rickettsial DNA from extracted products of each *Dermacentor* tick. Overall, six targeted partial fragments of genes were used (Table 2). Initially, detection and screening to determine the bacteria of the genus *Rickettsia* were carried out employing the genus-common 17-kDa surface antigen gene (17-kDa), the pan bacterial gene encoding 16S rRNA (16S rRNA) and the citrate synthase gene (*glt*A). *Rickettsia*-positive rates were then calculated. Additionally, we selected positive samples to test with other PCR amplifications that targeted the SFGRs-specific 190-kDa outer membrane protein A gene (*omp*A), the 120-kDa outer membrane protein B gene (*omp*B), and the PS120 or D protein-encoding gene (*sca*4) to determine their SFGRs features. The PCR amplicons of 17-kDa 434 bp*,* 16S rRNA 1,328 bp, *omp*A 532 bp, *omp*B 1,295 bp, and *sca*4 1,100 bp were purified using the NucleoSpin Gel and PCR Clean‑up Mini kit (MACHEREY–NAGEL, Germany). The purified amplicons were sequenced in both directions at Macrogen Co., LTD (Seoul, Korea). Furthermore, any ambiguous positive PCR products were gel purified, inserted into the pGEM® -T easy vector system (Promega, USA), and transformed into *E. coli* (strain JM109) using standard cloning protocols (or according to the manufacturer’s protocol). A positive cloning colony was grown on an LB-agar plate (Luria–Bertani) containing 20 mg/ml IPTG, 20 mg/ml X-Gal and 100 μg/ml ampicillin. The recombinant plasmids were also extracted and subjected to DNA sequencing.

### Data analysis

Data analyses of the obtained nucleotide sequences were primarily performed to determine the highest similarity using the Basic Local Alignment Search Tool (BLAST)^52^. Then, multiple sequence alignments with the related DNA sequences (Table S1 and S2) retrieved from the GenBank database were aligned by the CLUSTAL method, BioEdit v.2.0.0 program, while the amino acid polymorphisms were determined using the Amino Acid Explorer of the NCBI server^52^.

Phylogenetic trees were constructed using Neighbor-joining (NJ) method based on 16S rRNA and *COI* genes for tick species identification and Maximum-parsimony (MP) method based on 17-kDa, *glt*A, 16S rRNA, *omp*A, *omp*B, and *sca*4 genes for rickettsial genotype identification. These analyses were performed in the Molecular Evolutionary Genetics Analysis (MEGA) 7.0 software^53^. Distance matrix analysis was generated by the Tamura 3-parameters with gamma distribution (T92 + G) and Subtree-Pruning-Regrafting (SPR) for multiple substitutions. Bootstrap values were based on 1,000 replicates to estimate the support for nodes within the phylogenetic trees.

### Ethics approval

This research was approved by the SCMU-ACUC committee, protocol No. MUSC64-012-561.

## Supplementary Information

Supplementary Information.

## Data Availability

The nucleotide sequences of the *Rickettsia* obtained in this study are deposited to GenBank database (https://www.ncbi.nlm.nih.gov/genbank/).
