# Supplementary material for: A novel Rickettsia, Candidatus Rickettsia takensis, and the first record of Candidatus Rickettsia laoensis in Dermacentor from Northwestern Thailand
Source: Sci Rep. 2023 Jun 21;13:10044. doi: 10.1038/s41598-023-37206-w (PMC10284831; doi:10.1038/s41598-023-37206-w)
Supplement: Supplementary file 1 — Supplementary Information. [file 41598_2023_37206_MOESM1_ESM.pdf]

Supplementary information for: A novel *Rickettsia*, *Candidatus* Rickettsia takensis, and the first record of *Candidatus* Rickettsia laoensis in *Dermacentor* from northwestern Thailand

Aummarin Chaloemthanetphong[1], Arunee Ahantarig[1,4], Dmitry A. Apanaskevich[2], Supanee Hirunkanokpun[3], Visut Baimai[1] & Wachareeporn Trinachartvanit[1✉]

[1]Biodiversity Research Cluster, Department of Biology, Faculty of Science, Mahidol University, Bangkok 10400, Thailand.

[2]United States National Tick Collection, the James H. Oliver, Jr. Institute for Coastal Plain Science, Georgia Southern University, Statesboro, GA 30460-8056, USA.

[3]Department of Biology, Faculty of Science, Ramkhamhaeng University, Bangkok 10240, Thailand.

[4]Center of Excellence for Vectors and Vector-Borne Diseases, Faculty of Science, Mahidol University at Salaya, Nakhon Pathom 73170, Thailand.

✉email: wachareeporn.tri@mahidol.ac.th

Species (Country)	Code	GenBank accession no.					
		**17-kDa**	**16S rRNA**	***glt*A**	***omp*A**	***omp*B**	***sca*4**
R. fournieri (Australia)	AUS118	NZLT978484	KF666475	KF666471	KF666477	LT978484	KF666473
R. japonica (China)	LA16/2015	CP047359	CP047359	CP047359	CP047359	CP047359	CP047359
Ca. R. vini (The Czech Republic)	Breclav	KT187396	NA	KT187394	KT326194	NA	NA
Rickettsia sp. (Australia)	ARRL2016-159	MN431839	NA	MN431836	MN431847	NA	NA
Ca. R. xinyangensis (China)	XY118	KY617773	NA	KY617774	KY617775	NA	KY617777
R. japonica (Japan)	YHM	AP017602	AP017602	AP017602	AP017602	AP017602	AP017602
R. japonica (Thailand)	PMK94	DQ909071	NA	DQ909073	DQ909072	NA	NA
R. japonica (Thailand)	TCM1	AB359457	NA	AB359458	AB359459	NA	NA
R. japonica (Japan)	YM	NA	L36213	U59724	U43795	AF123713	AF155055
R. heilongjiangensis (Japan)	Sendai-58	AP019865	AP019865	AP019865	AP019865	AP019865	AP019865
Rickettsia sp. (Laos)	315	KT753267	NA	KT753268	NA	KT753270	KT753269
Rickettsia sp. (Laos)	76	KT753277	NA	KT753278	NA	KT753280	KT753279
Rickettsia sp. (Laos)	MT55-R	LC456207	NA	LC456206	NA	NA	NA
Rickettsia sp. (Japan)	Hys 2024	LC379445	LC379484	LC379428	NA	NA	NA
Rickettsia sp. (USA)	DaE100R	NA	AF097729	AF129885	NA	NA	NA
Ca. R. laoensis (Laos)	447	KT753291	NA	KT753290	KT753293	KT753294	KT753292
Ca. R. laoensis (China)	MHS 2019/3	NA	NA	NA	MT321615	NA	NA
Ca. R. laoensis (Taiwan)	Da-1	NA	NA	MZ869826	MZ869827	MZ869829	MZ869830
Rickettsia sp. (India)	MIVLW15/2017	MN557230	NA	MN557219	MK905251	NA	NA
Rickettsia sp. (Thailand-Myanmar border)	RDla440	NA	NA	AF497585	NA	NA	NA
Rickettsia sp. (Pakistan)	JC880	NA	MN577234	NA	NA	NA	NA
Ca. R. takensis (Thailand)	DLT9F1.1	ON704036	ON705030	ON704040	ON704044	ON704052	ON730890
Ca. R. takensis (Thailand)	DLT9M1	ON704037	ON705031	ON704041	ON704045	ON704053	ON730891
Ca. R. takensis (Thailand)	DSNT11M3.2	ON704038	ON705032	ON704042	ON704046	ON704054	ON730892
Ca. R. laoensis (Thailand)	DAT10M3	ON704039	ON705033	ON704043	ON704047	ON704055	ON730893

Table S1. Accession numbers of gene sequences of *Rickettsia* spp. used in the study. *NA* not available.

**Species (Country)**	**Code**	**GenBank accession no.**	
		**16S rRNA**	***COI***
D. auratus (Thailand)	DAT02	ON705024	ON680798
D. auratus (Thailand)	DAT03	ON705025	ON680799
D. auratus (Malaysia)	VGBb	MZ005646	MW971473
D. auratus (Malaysia)	VGATS2b	MZ005648	MW971475
D. auratus (Singapore)	TKL1	MT371591	MT371767
D. albipictus (USA)	1	ON800833	ON800833
D. andersoni (USA)	OSU15429 DandMT M9	NC061057	NC061057
D. compactus (Malaysia)	VGATS1e	MZ005650	MW971491
D. everestianus (China)	18	KJ599808	KJ599815
D. falsosteini (Malaysia)	VGBg	MZ005641	MW971501
D. laothaiensis (Thailand)	DLT02	ON705026	ON680800
D. laothaiensis (Thailand)	DLT03	ON705027	ON680801
D. marginatus (China)	E1	NC062069	NC062069
D. nitens (Brazil)	Deni	NC023349	NC023349
D. reticulatus (Russia)	NA	MT478096	MT478096
D. rhinocerinus (South Africa)	76/110 14708 43	KY457526	KY457526
D. silvarum (China)	NA	KP258209	KP258209
D. steini (Thailand)	DST01	ON705028	ON680802
D. steini (Thailand)	DST02	ON705029	ON680803
D. steini (Malaysia)	VGATS1f	MZ005639	MW971469
D. steini (Malaysia)	HPVa	MZ005637	MW971470
D. steini (Malaysia)	VGATS2a	MZ005640	MW971472
D. variabilis (USA)	DvarOH F100	MN190610	MN190610

Table S2. Accession numbers of gene sequences used in the study of concatenated genes. *NA* not available.

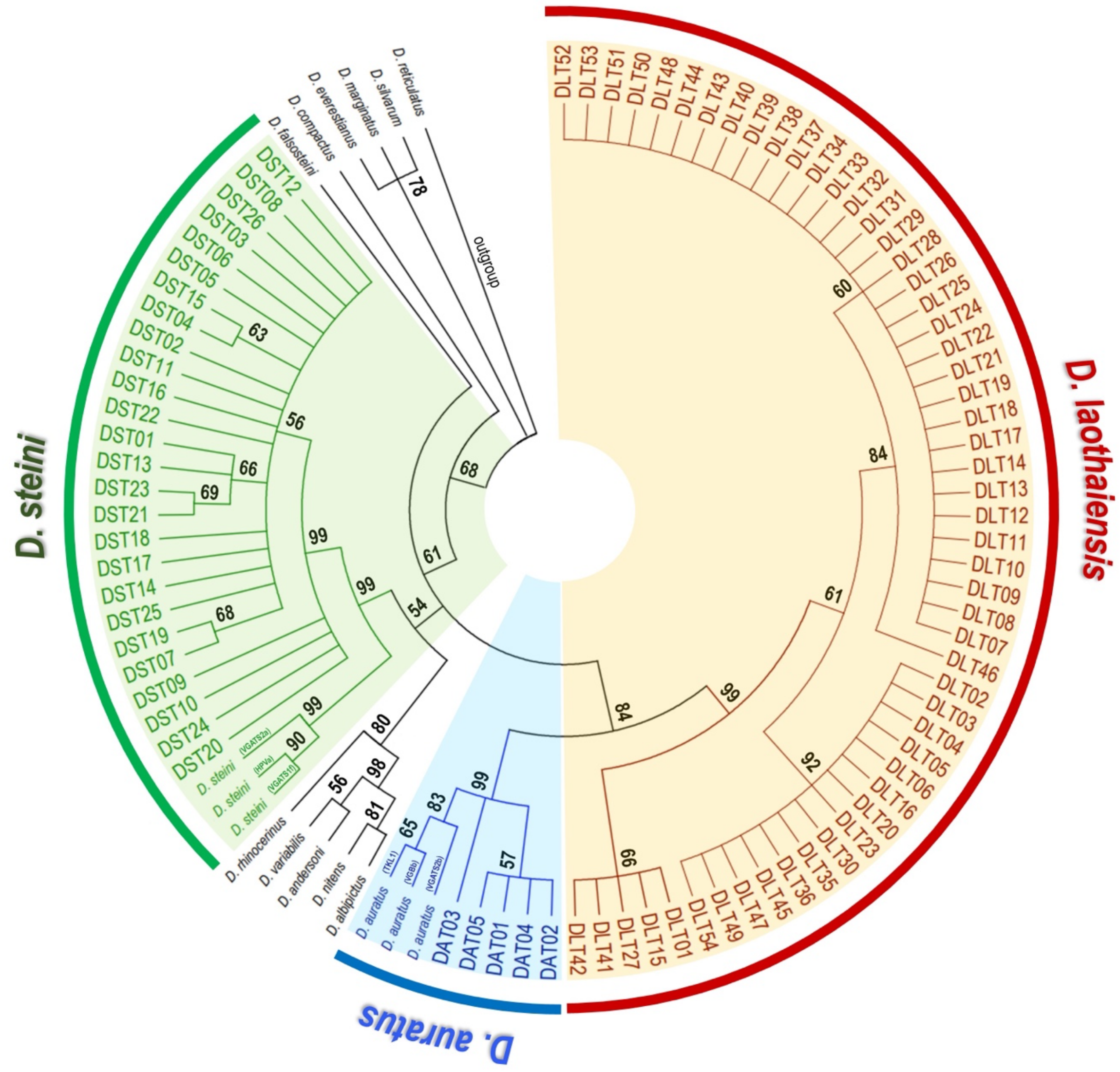

Figure S1. Phylogenetic tree constructed using neighbor-joining (NJ) estimations based on concatenated 16S rRNA and *COI* sequences (best-fit approximation for the evolutionary model: Tamura 3-parameter method and gamma distribution). The tree represents three different species of the *Dermacentor* ticks from Tak province; *D. laothaiensis* (DLT), *D. steini* (DST), and *D. auratus* (DAT). The reference species for *D. steini* and *D. auratus* are included with their accession numbers, while *D. laothaiensis* shows the first molecular identification. The outgroup was *D. reticulatus* (MT478096). Only bootstrap values >50% are shown above each branch.

16S rRNA

Tick species — Position of nucleotide/amino acid (5'→3')

Tick species	281	398
D. auratus (DAT03) Thailand	G	-
D. auratus Thailand	A	T

	217	258
D. auratus (DAT03) Thailand	G	T
D. auratus (voucher PKY016-04) Malaysia	A	A

	14	33	84	124	126	164	197	199	201	202	203	212	213	214	216	218	400
D. steini (DST02) Thailand	-	T	-	A	A	C	G	A	T	T	A	T	A	A	T	C	T
D. steini (Eng 56) Malaysia	A	C	A	G	T	A	A	T	A	A	C	G	T	T	A	T	A

	7	9	33	114	119	124	126	151	152	165	176	188	191	194	199	200	205	206	208	211	217	218	223	226	227 →
D. laothaiensis (DLT03) Thailand	C	T	T	-	G	A	A	A	A	A	G	C	T	A	A	C	A	T	T	A	G	T	T	A	C
D. steini strain (Eng 56) Malaysia	T	A	C	T	T	G	T	T	T	T	A	T	A	T	T	T	-	-	A	T	A	A	A	T	A

	→ 251	259	314	315	319	325	335
	A	T	T	T	T	A	T
	T	A	C	A	C	G	C

	7	9	25	77	84	120	145	150	151	153	190	194	200	201	203	205	206	207	209	210	214	215	220	226	227 →
D. laothaiensis (DLT03) Thailand	C	T	T	A	A	C	T	A	A	A	T	-	C	A	C	A	T	A	A	T	G	T	T	A	C
D. auratus (DAT03) Thailand	T	G	C	T	T	T	G	T	T	T	A	T	T	T	A	T	A	C	T	A	A	A	-	T	T

	→ 228	251	282	289	292	302	319	323
	A	A	A	T	T	A	T	A
	T	C	G	C	C	T	C	T

	6	84	114	119	156	157	164	165	176	188	191	194	197	200	201	202	205	209	211	214	215	216	217	218	219 →
D. laothaiensis (DLT03) Thailand	C	A	-	G	A	A	A	A	G	C	T	A	A	-	-	C	C	A	A	T	T	G	T	T	G
D. steini (DST02) Thailand	-	-	T	T	T	T	C	T	A	T	A	T	G	T	T	T	A	T	T	-	-	-	-	A	A

	→ 222	225	228	229	257	261	316	317	321	327	337
	T	T	A	C	A	T	T	T	T	A	T
	C	A	T	A	T	A	C	A	C	G	C

	9	25	77	84	118	121	146	154	164	165	176	188	189	198	203	206	208	211	212	214	218	219	223	227	228 →
D. steini (DST02) Thailand	-	T	A	-	T	C	T	A	C	T	A	T	-	G	T	A	A	T	T	A	A	C	-	A	A
D. auratus (DAT03) Thailand	G	C	T	T	G	-	G	T	A	A	G	C	A	A	A	T	C	A	A	T	G	T	T	T	T

	→ 251	259	282	289	292	302	314	315	323	325	335
	T	A	A	T	T	A	C	A	A	G	C
	C	T	G	C	C	T	T	T	T	A	T

COI

Tick species — Position of nucleotide/amino acid (5'→3')

Tick species	2	5	47	188	284
D. auratus (DAT02) Thailand	T	C	A	C	C
D. auratus (VGBb) Malaysia	A	T	G	A	A

	2	5	8	36	44	47	48	57	68	83	92	101	113	116	125	137	146	170	182	185	230	248	320	338
D. steini (DST02) Thailand	T	C	T	C	A	T	T	C	T	G	A	G	C	T	A	C	G	T	A	C	T	T	A	T
D. steini (VGATS2a) Malaysia	A	T	C	T	T	C	C	T	C	A	G	A	A	C	C	T	A	C	G	T	C	C	G	C

	20	32	35	38	41	53	57	62	68	71	77	92	128	134	140	158	164	167	170	188	191	203	221	230	242	244	248	251	254 →
D. laothaiensis (DLT03) Thailand	C	G	A	T	A	T	C	A	A	C	C	A	T	T	C	A	T	T	T	T	A	G	T	A	C	T	A	G	A
D. auratus (DAT02) Thailand	T	A	T	C	T	C	T	T	T	A	T	G	C	G	T	T	C	C	C	C	T	A	C	C	T	C	T	A	T

	→ 260	263	270	272	284	305	308	311	335	338	348	365	374
	G	G	C	G	A	T	C	G	T	C	T	T	T
	A	A	T	A	C	C	T	A	A	A	C	C	A

	20	32	33	38	41	44	47	60	68	71	77	83	101	113	122	134	140	143	146	149	155	156	171	173	176	177	182	185	188 →
D. laothaiensis (DLT03) Thailand	C	G	C	T	A	C	A	G	A	C	C	A	A	T	T	T	C	C	T	A	T	C	C	T	C	T	T	T	T
D. steini (DST02) Thailand	T	A	T	A	C	A	T	A	T	A	T	G	G	C	A	A	T	T	G	C	C	T	T	A	T	C	A	C	A

	→ 191	230	242	248	251	254	260	263	270	272	287	309	311	338	344	356	374
	A	A	C	A	G	A	G	G	C	G	C	T	G	C	T	A	T
	C	T	A	T	A	T	A	T	T	A	T	C	T	T	C	T	C

	2	5	8	20	32	33	36	38	41	44	47	48	57	60	68	71	77	92	113	116	122	125	134	137	140	143	146	149	155 →
D. laothaiensis (DLT03) Thailand	T	C	T	C	G	C	C	T	A	C	A	T	C	G	A	C	C	A	T	T	T	A	T	C	C	C	T	A	T
D. steini (VGATS2a) Malaysia	A	T	C	T	A	T	T	A	C	T	C	C	T	A	C	A	T	G	G	C	A	C	A	T	T	T	A	C	C

	→ 156	170	171	173	176	177	182	188	191	230	242	248	251	254	260	263	270	272	287	309	311	320	344	356
	C	T	C	T	C	T	T	T	A	A	C	A	G	A	G	G	C	G	C	T	G	A	T	A
	T	C	T	C	T	C	G	A	C	C	A	C	A	T	A	T	T	A	T	C	T	G	C	T

	33	35	38	41	44	47	53	57	60	62	83	92	101	113	122	128	134	143	146	149	155	156	158	164	167	170	171	173	176 →
D. steini (DST02) Thailand	T	A	A	C	A	T	T	C	A	A	G	A	G	C	A	T	A	T	G	C	C	T	A	T	T	T	T	A	T
D. auratus (DAT02) Thailand	C	T	C	T	C	A	C	T	G	T	A	G	A	T	T	C	G	C	T	A	T	C	T	C	C	C	C	T	C

	→ 177	182	185	188	191	203	221	230	242	244	263	284	287	305	308	309	311	335	338	344	348	356	365	374
	C	A	C	A	C	G	T	T	A	T	T	A	T	T	C	C	T	T	T	C	T	T	T	C
	T	T	T	C	T	A	C	C	T	C	A	C	C	C	T	T	A	A	A	T	C	A	C	A

Figure S2. Nucleotide sequence alignments of the related *Dermacentor* tick species based on the partial fragments of the variable genes 16S rRNA and *COI*. The comparisons represented different nucleotide base positions and dashes (-) demonstrated gap positions.

17-kDa

Rickettsia spp.	Position of nucleotide/amino acid (5'→3')	
	173	363
Ca. R. takensis (DLT9M1) Thailand	A	T
	Lys	Tyr
Ca. R. vini (Breclav) The Czech Republic	G	C
	Arg	Tyr

	173	363
Ca. R. takensis (DLT9M1) Thailand	A	T
	Lys	Tyr
Rickettsia sp. (TCMI) Thailand	G	C
	Arg	Tyr

	173	363
Ca. R. takensis (DLT9M1) Thailand	A	T
	Lys	Tyr
R. fournieri (AUS118) Australia	G	C
	Arg	Tyr

16Sr RNA

Rickettsia spp.	Position of nucleotide/amino acid (5'→3')						
	19	75	498	544	789	965	977
Ca. R. takensis (DLT9M1) Thailand	-	T	G	G	T	A	T
R. fournieri (AUS118) Australia	A	G	A	A	C	G	C

	19	29	75	544	789	965	977
Ca. R. takensis (DLT9M1) Thailand	-	C	T	G	T	A	T
R. japonica (LA16/2015) China	A	T	G	A	C	G	C

	19	29	75	544	789	965	977
Ca. R. takensis (DLT9M1) Thailand	-	C	T	G	T	A	T
R. japonica (YHM) Japan	A	T	G	A	C	G	C

	19	29	75	544	789	965	977
Ca. R. takensis (DLT9M1) Thailand	-	C	T	G	T	A	T
R. japonica (YM) Japan	A	T	G	A	C	G	C

	19	29	75	544	789	965	977	1055
Ca. R. takensis (DLT9M1) Thailand	-	C	T	G	T	A	T	C
R. heilongjiangensis (Sendai-58) Japan	A	T	G	A	C	G	C	T

***glt*A**

Rickettsia spp.	Position of nucleotide/amino acid (5'→3')
	171
Ca. R. takensis (DLT9M1) Thailand	A
	Thr
R. japonica (PMK94) Thailand	G
	Thr

	171
Ca. R. takensis (DLT9M1) Thailand	A
	Thr
Rickettsia sp. (ARRL2016-159) Australia	G
	Thr

	168
Ca. R. takensis (DLT9M1) Thailand	A
	Thr
R. heilongjiangensis (Sendai-58) Japan	G
	Thr

***omp*A**

Rickettsia spp.	Position of nucleotide/amino acid (5'→3')														
	79	94	139	142	153	154	165	166	167	221	227	282	325	331	332
Ca. R. takensis	G	A	G	G	A	A	-	-	-	A	T	T	G	A	A
	Val	Asn	Val	Ala	Gly	Ile	-	-	-	Asn	Ile	Pro	Val	Lys	Lys
R. fournieri strain AUS118 Australia	A	G	A	A	G	G	T	A	C	C	C	C	A	G	G
	Ile	Asp	Ile	Thr	Gly	Val	Gly	Thr	Thr	Thr	Thr	Pro	Ile	Gly	Gly

***omp*B**

Rickettsia spp.	Position of nucleotide/amino acid (5'→3')																				
	139	259	319	467	527	532	621	697	741	795	977	1062	1065	1068	1069	1071	1072	1073	1074	1075	1076
Ca. R. takensis (DLT9M1) Thailand	T	A	C	T	T	A	G	A	T	T	A	T	T	A	G	-	-	-	-	-	-
	Leu	Asn	His	Ile	Ile	Asn	Gly	Ser	Gly	Gly	Tyr	Thr	Ile	Gly	Ala	-	-	-	-	-	-
R. fournieri (AUS118) Australia	G	G	A	C	C	G	A	G	C	C	G	A	C	T	A	T	A	T	T	G	G
	Val	Asp	Asn	Thr	Thr	Asp	Gly	Gly	Gly	Gly	Cys	Thr	Ile	Gly	Thr	Thr	Ile	Ile	Ile	Gly	Gly

***sca*4**

Rickettsia spp.	Position of nucleotide/amino acid (5'→3')										
	138	233	242	426	516	686	856	871	916	930	942
Ca. R. takensis (DLT9M1) Thailand	C	G	G	A	G	T	A	T	T	T	C
	Tyr	Val	Gly	Thr	Met	Ile	Thr	Tyr	Tyr	Val	Ser
R. fournieri (AUS118) Australia	T	A	A	G	T	C	G	C	G	C	T
	Tyr	Ile	Glu	Thr	Ile	Thr	Ala	His	Asp	Val	Ser

Figure S3. Alignments of nucleotide and amino acid sequences of *Ca.* R. takensis compared with the related species of *Rickettsia*. The comparisons show different nucleotide base positions and dashes (-) demonstrated gap with their translated amino acids for 17-kDa, *glt*A, *omp*A, *omp*B, and *sca*4 except for 16S rRNA which does not have amino acid. The variations of amino acids are highlighted.
